# Implication of metastasis suppressor gene, Kiss-1 and its receptor Kiss-1R in colorectal cancer

**DOI:** 10.1186/1471-2407-14-723

**Published:** 2014-09-27

**Authors:** Ke Ji, Lin Ye, Fiona Ruge, Rachel Hargest, Malcolm D Mason, Wen G Jiang

**Affiliations:** Cardiff University-Peking University Joint Oncology Institute, Metastasis & Angiogenesis Research Group, Cardiff University School of Medicine, Cardiff, CF14 4XN UK; Section of Oncology & Palliative Medicine, Cardiff University School of Medicine Velindre Hospital, Cardiff, CF14 2TL UK

**Keywords:** Metastasis suppressor gene, Kiss-1, Kiss-1R, GPR54, Colorectal cancer, Migration, Invasion, MMP-9, ERK signal pathway

## Abstract

**Background:**

Kiss-1 and Kiss-1R have been suggested as a novel pair of metastasis suppressors for several human solid tumours, however, their role in colorectal cancer remains largely unknown. Therefore, the aim of this study was to investigate the role and signal transduction of Kiss-1 and Kiss-1R in colorectal cancer.

**Methods:**

Ribozyme transgenes were used to knockdown high expression of Kiss-1 and Kiss-1R in HT115 and HRT18 cells. The stabilized transfected cells were then used to deduce the influence of Kiss-1 and Kiss-1R on the function of colorectal cancer cells by *in vitro* assays and ECIS assay. The effect of Kiss-1 on MMPs related to tumour metastasis was also deleted by zymography. The mRNA and protein expression of Kiss-1 and Kiss-1R, and their correlation to the clinical outcome in human colorectal cancer were investigated using real-time PCR and IHC respectively.

**Results:**

Knocking down Kiss-1 resulted in increased invasion and migration of colorectal cancer cells. Kisspeptin-10 decreased cellular migration of colorectal cancer cells and required ERK signaling as shown during the ECIS based analyses. Reduction of MMP-9 was caused by Kisspeptin-10 and ERK inhibitor, shown by zymography. In human colorectal cancer tissues, the mRNA expression level of Kiss-1 had a negative correlation with Dukes staging, TNM staging, tumour size and lymph node involvement. Reduction of Kiss-1R was also linked to poor prognosis for the patients.

**Conclusions:**

The present study has presented evidence that Kiss-1 may be a putative metastasis suppressor molecule in human colorectal cancer.

## Background

Colorectal cancer (CRC) is the second most commonly diagnosed cancer and is a major cause for mortality and morbidity globally [1].

The *Kiss-1* gene was identified as a human melanoma metastasis suppressor gene through the analysis of subtractive hybridization in highly metastatic cell lines as compared to non-metastatic cell lines [2]. The *Kiss-1* gene encodes a protein of 145-amino-acids, which is subsequently cleaved into a family of Kisspeptins, including Kisspeptin-10, Kisspeptin-13, Kisspeptin-14, Kisspeptin-54 respectively [3–5]. Its receptor, Kiss-1R, also known as G-protein coupled receptor 54 (GPR54) was first discovered and cloned from rat brain in 1999 [6].

Kiss-1 and Kiss-1R have been suggested as a novel pair of metastasis suppressors in most cancers [5, 7–11]. Correlation between decreased expression of Kiss-1 and poor clinical outcomes has been evident in most malignancies that have been investigated. One possible explanation for the role played by Kiss-1 in cancer biology could be extrapolated from the relationship between Kiss-1 and matrix metalloproteinases (MMPs), whose significance in tumour invasion and metastasis formation is well known [12]. However, few studies have specifically shown a role for Kiss-1 in colorectal cancer as yet.

In this study, we examined the expression of Kiss-1 and Kiss-1R in human colorectal cancer and analyzed the potential clinical and prognostic implications. After that, we investigated their effect on the function of colorectal cancer cells. On the basis of the data from these experiments, Kiss-1 may play a metastasis suppressor role in human colorectal cancer and be linked to the disease progression of patients, by way of aberrant expression and molecular and cellular mechanism (s) that are yet to be identified.

## Methods

### Patients and tissue specimens

Colorectal cancer tissues (n = 94) and normal background tissues (n = 80) were collected immediately after surgery with approval by the South East Wales Local Research Ethics Committee (ref 05/WSE03/92) and patients’ consent. The tissue samples were stored in a deep freezer (−80°C) until further use. Patients were routinely followed after surgery and the median follow up period was 120 months. Tumour tissues and normal tissues (>10 cm away from the tumor margin) were obtained with confirmation by a pathologist.

### Cell culture

HT115 and HRT18 cancer cell lines were obtained from the European Collection of Animal Cell Culture (ECACC, Salisbury, England, UK).

### Peptide receptors and inhibitors, antibodies

ERK inhibitorII (FR180204) (Calbiochem, Germany), Kisspeptin-10 (No.2570) (Tocris Bioscience, Bristol, UK) and Kisspeptin-234 (Tocris Bioscience, Bristol, UK). Kisspeptin-10, a short peptide of 10 amino acids, is proteolytically processed from Kiss-1 [13]. Kisspeptin-234 is a Kisspeptin-10/Kiss-1R antagonist, which belongs to Kisspeptin-10 analog. Antibodies to Kiss-1 (SC-101246) and Kiss-1R (SC-48220) were purchased from Santa Cruz Biotechnologies Inc., (Santa Cruz, CA, USA).

### RNA isolation, cDNA synthesis, RT-PCR, Q-PCR and immunohistochemical staining

RNA extraction kits, reverse transcription kits and RT-PCR Mix were purchased from Promega (WI, USA) and Bio-Rad (CA, USA). Conventional PCR primers were designed using Beacon Designer (Palo Alto, CA, USA) and synthesized by Invitrogen (Paisley, Scotland, UK). Following the manufacturer’s protocol, total RNA was isolated using TRI reagent (Sigma). The concentration of RNA was detected by a UV spectrophotometer at 260 and 280 nm. cDNA samples were synthesized and the final reaction volume was 20μl. Primers used for RT-PCR are given in Table 1, and GAPDH was used as the house keeping control.

**Table 1:** **Primer sequences used for RT-PCR and Q-PCR in this study**

Genes		Sense (5'-'3)	Antisense (5'-'3)
**Kiss-1**	Conventional PCR	TGAACTCACTGGTTTCTTGG	CGAAGGAGTTCCAGTTGTAG
	Quantitative PCR	CATTAGAAAAGGTGGCCTCT	ACTGAACCTGACCGTACAGCCCAGGGATTCTAGCTG
	Anti-Kiss1 ribozyme1	CTGCAGCTCTCGGGGGGCGGGGACAGCGAGGTCCCCCCTGATGAGTCCGTGAGGA	ACTAGTGCCAGCTGCTACTGCCAGGCTGAGCCGTTTCGTCCTCACGGACT
	Anti-Kiss-1 ribozyme2	CTGCAGCACCGCGCCCTGGGGTGCGGGCTGATGAGTCCGTGAGGA	ACTAGTGTCCGCCCCCCACAGCCGCCAGATTTCGTCCTCACGGACT
**Kiss-1R**	Conventional PCR	CTTCATGTGCAAGTTCGTC	CACCAGGAACAGCTGGAT
	Quantitative PCR	GCTGGTCATCTACGTCATCT	ACTGAACCTGACCGTACACAGCACAGGAGGAAGGTC
	Anti-Kiss-1R ribozyme1	CTGCAGTTCCGCATCGGCTTGTGGCGGCACTGATGAGTCCGTGAGGA	ACTAGTTCGCTGGTCATCTACGTCATTTCGTCCTCACGGACT
	Anti-Kiss-1R ribozyme2	CTGCAGAGCCTACCCAGATGCTGAGGCTCTGATGAGTCCGTGAGGA	ACTAGTGCCCCGCCTGGCGCTGGCTGTTTCGTCCTCACGGACT
**GAPDH**	Conventional PCR	GGCTGCTTTTAACTCTGGTA	GACTGTGGTCATGAGTCCTT
	Quantitative PCR	CTGAGTACGTCGTGGAGTC	ACTGAACCTGACCGTACACAGAGATGATGACCCTTTTG

**Table 4:** **Multivariate analysis for prognostic factors for colorectal related death and incidence** (***p*** **values)**

Variables	With colorectal related death	With colorectal related incidence
Dukes stage	0.507	0.249
T-stage	0.025	0.092
TNM stage	0.146	0.030
Node status	0.230	0.057
Differentiation	0.601	0.326
Kiss-1	0.435	0.566
Kiss-1R	0.003	0.012

Real-time quantitative PCR, based on the Amplifluor™ technology, was used to quantify the level of mRNA expression of Kiss-1 and Kiss-1R from the cDNA samples of coloretal tissues and cells, mentioned above. All colorectal cDNA samples were synchronously examined for Kiss-1 and Kiss-1R along with a set of internal controls. Q-PCR primers (Table 1) were designed using Beacon Design software (PREMIER Biosoft, Palo Alto, CA). Real-time PCR was carried out using an IcyclerIQ™ (Bio-Rad, Hemel Hempstead, UK) with the following cycling conditions: 94°C for 5 min, 80–90 cycles of: 94°C for 10 sec, 55°C for 35 sec and 72°C for 20 sec.

Frozen sections of colorectal tumours and adjacent background tissues were sectioned at a thickness of 6 μm using a cryostat . The samples were mounted onto Super Frost Plus microscope slides (Fisher, UK). The samples were fixed in a mixture of 50% acetone and 50% methanol and then air-dried. After rehydration and blocking with 5% horse serum solution, the sections were probed with the appropriate primary antibody and secondary antibodies. Following the instructions, the avidin-biotin complex (Vector Laboratories, Burlingame, CA, USA) was applied before staining with diaminobenzidine chromogen. Nuclei were counterstained in Mayer’s haematoxylin. Sections from fresh frozen human placenta were used as positive controls.

### Generation of Kiss-1 and Kiss-1R ribozyme transgenes and stable transfectants

**Figure 1:**
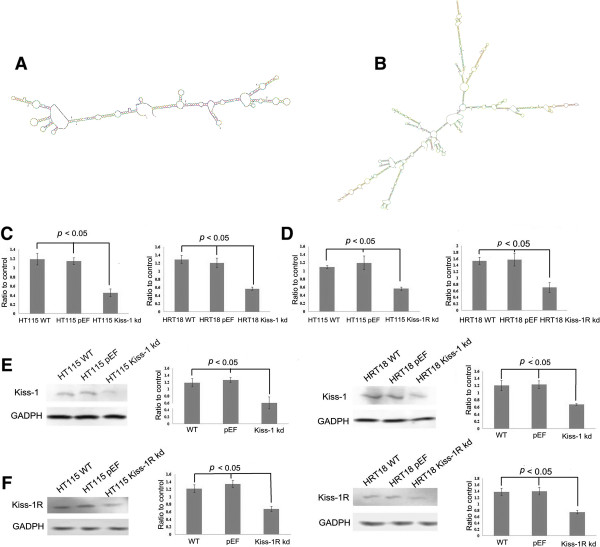
**Genetic modification of Kiss-1 and Kiss-1R expression in colon cancer cells.** A and B: Secondary structures of Kiss-1 **(A)** and Kiss-1R **(B)** used to design anti-Kiss1 and Kiss-1 ribozymes C and D: Quantitative real time PCR showed Kiss-1 **(C)** and Kiss-1R **(D)** mRNA volume of three repeats which was normalized against corresponding internal control (GAPDH). E and F: Confirmation of Kiss-1 **(E)** and Kiss-1R **(F)** knockdown in HT115 cells and HRT18 cells using Western blot.

Hammerhead ribozymes targeting Kiss-1 and Kiss-1R were designed using Zuker’s mRNA Fold programme [14] based on the secondary structure of Kiss-1 and Kiss-1R mRNA (Figure 1A and B, respectively). The ribozymes were synthesized using touchdown PCR and cloned into the pEF6/V5-His TOPO TA expression plasmid vector (Invitrogen, Paisley, UK) according to the protocol provided. Ribozyme transgenes and empty plasmids were transfected into the two colorectal cell lines HT115 and HRT18 respectively, utilizing an Easyjet Plus electroporator (EquiBio, Kent, United Kingdom). Following selection using blasticidin, verified transfectants which lost the expression of Kiss-1 and Kiss-1R were used in subsequent experiments. During these experiments, cDNA generated from human placenta was used as a positive control.

### *In vitro*cell function assays

#### In vitro cell growth assay

Cells were seeded into 96-well plates at 2,500 cells/well, and cultured using normal media (10% fetal calf serum, 0.1% antibiotics). The cells were cultured in triplicate for 1, 3 and 5 days. After incubation the cells were fixed in 4% formalin and stained with 0.5% crystal violet (w/v). The stained crystal violet was then extracted using 10% (v/v) acetic acid, and the absorbance was determined using a spectrophotometer (Bio-Tek, ELx800) at a wavelength of 540 nm.

#### In vitro cell adhesion assay

A 96-well plate was precoated with 5 μg of Matrigel (CollaborativeResearch Products, Bedford, Massachusetts, USA) and allowed to air dry. Following rehydration using serum free media, 40,000 cells were seeded into each well. After 40 minutes of incubation, non-adherent cells were washed off using BSS. The adherent cells were then fixed with 4% formalin and stained using 0.5% crystal violet. The number of adherent cells were counted under a microscope.

#### In vitro invasion assay

Transwell inserts (with 8 μm pore) were precoated with 50 μg of Matrigel and air dried. Following rehydration, 40,000 cells were seeded into each insert. After incubation for three days, cells which had invaded through the matrix and adhered to the other side of the insert were fixed in 4% formalin, and stained with 0.5% (w/v) crystal violet. The number of invaded cells was then counted under a microscope.

#### In vitro wounding assay for cellular migration

Cells were seeded into a 24-well plate at a density of 200,000 per well and allowed to form a monolayer of cells. The monolayer of cells was then scraped to create a wound. Migration of cells at the wound edges were monitored over a period up to 18 hours. Optimas 6.0 motion analysis (Meyer Instruments, Houston, Texas) was used to track the leading edge of cells to measure the distance of migration.

### Electric cell-substrate impedance sensing (ECIS)

The ECIS system (Ztheta, Applied Biophysics Inc., USA) was used to quantify cell migration as previously reported by Jiang *et al.* [15]. HT115 cells were prepared for six repeats per group and seeded at 40,000 cells per well in 200 μl of DMEM medium alone or medium supplemented with 200 nM ERK small inhibitor, 300 nM Kisspeptin-10 and 300 nM Kisspeptin-234.

### Gelatin zymography assay

Cells were counted and 1×10^6^ cells were seeded to a tissue culture flask. After an overnight incubation and 4 hours treatment with appropriate peptide receptor or inhibitor (300 nM Kisspeptin-10, 300 nM Kisspeptin-234 or 200 nM ERK inhibitor), the medium was collected. This method is well established within the laboratory, and has previously been published [16].

### Data analysis

The relationship between the expression of Kiss-1 and Kiss-1R and tumour grade, TNM staging and nodal status was respectively analyzed using Mann–Whitney U test (Tables 2 and 3). Quantitative data of IHC, *in vitro* assays, ECIS assay and zymography assay was analyzed using the Student’s test, Kruskal-Wallis and chi-squared test, where appropriate. Survival analysis and multivariate analysis were carried out using the SPSS20 software package. Differences were considered to be statistically significant at p *<* 0.05.

## Results and discussion

### Kiss-1 and Kiss-1R expression in colorectal adenocarcinoma tissues and the histopathological/clinical characteristics of the disease

Kiss-1 and Kiss-1R expression was analysed in colorectal cancer tissues and adjacent normal tissues using real time PCR (Tables 2 and 3). Decreased levels of Kiss-1 transcript were seen in Dukes B and C tumours compared with Dukes A carcinomas (*p* < 0.05). The expression level of Kiss-1 decreased as TNM stage progressed, and statistical analysis revealed significant links between TNM stage I and stage III&IV. A significantly decreased expression of Kiss-1R was observed in tumour tissues compared with normal background tissues. Interestingly, Kiss-1R expression in patients undergoing chemo-radio therapy was considerably higher in comparison to that in patients without therapy.

**Figure 2:**
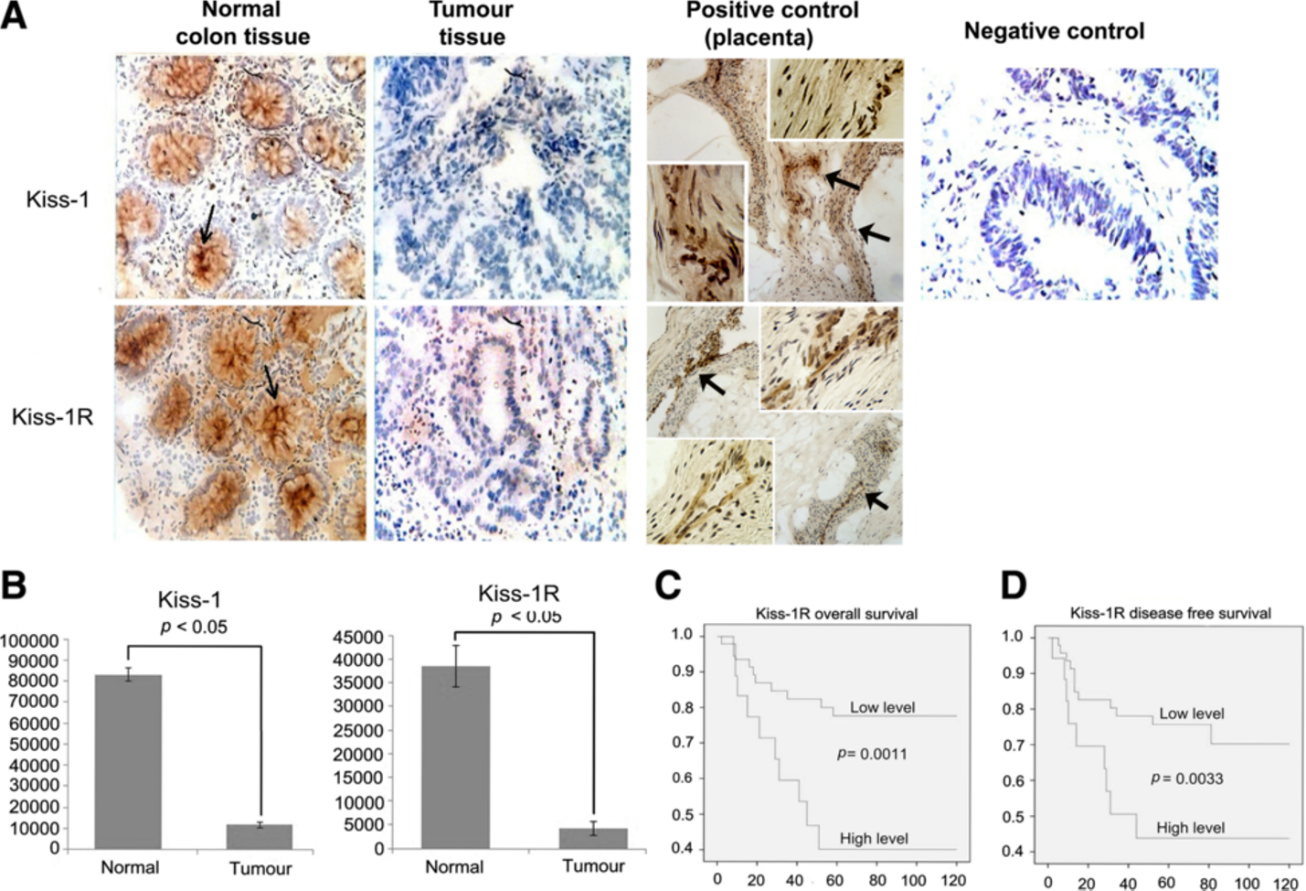
**Expression of Kiss-1 and Kiss-1R in colorectal cancer. A**. Immunohistochemical staining of Kiss-1 and Kiss-1R in normal colorectal tissue and tumour tissue; Arrows point to the staining of Kiss-1 and Kiss-1R in the cytoplasm of normal cells. Placenta was used as the positive control for both Kiss-1 and Kiss-1R. **B**. Semi quantitative analysis used to verify the protein expression level of Kiss-1 and Kiss-1R protein in normal background and tumour tissue. **C**. Kaplan- Meier survival analysis displaying relationship between the transcript levels of Kiss-1R and overall survival. **D**. Kaplan- Meier survival analysis displaying the relationship between the transcript levels of Kiss-1R and disease free survival. Patients with high levels of Kiss-1R had a significantly shorter overall survival being 85.8 months (95% C.I. 49.9- 121.6 months), *p* = 0.011 in comparison to those with low levels (143.0 months, 95% C.I. 124.261- 161.712). The patients with lower expression of Kiss-1R also had longer disease free survival (median = 133.2 months, 95% C.I. 111.7- 154.7 months), *p* = 0.033 compared with those with higher expression levels (median = 88.3 months, 95% C.I. 50.1- 126.6).

A significant difference was also observed in the expression levels between living patients and those who had died. The average copy numbers of Kiss-1 and Kiss-1R transcripts in Dukes B were then employed as the respective thresholds for the survival analysis. Kaplan-Meier survival analysis demonstrated that patients with a low expression level of Kiss-1 appeared to have similar overall survival and disease free survival compared to patients with high expression of Kiss-1 (*p* > 0.05). In contrast to Kiss-1, the expression pattern of Kiss-1R revealed that high levels of Kiss-1R transcript are associated with both a poor overall survival (Figure 2C, p = 0.0011) and poor disease free survival (Figure 2D, p = 0.0033). Multivariate analyses have further demonstrated that T-stage and Kiss-1R are independent prognostic factors (p = 0.025 and p = 0.003, respectively) for colorectal related death. Furthermore, TNM staging and Kiss-1R (p = 0.03 and p = 0.012, respectively) are independent prognostic factors for colorectal cancer related incidence (death, recurrence and metastasis) (Table 4).

**Table 2:** **The correlation of mRNA expression of Kiss-1 and clinical parameters**

Category		No.	Median	IQR	***p***	***P*** ^a^
T/N^b^						
	Normal	80	97	6-2345		
	Tumour	94	35	1-2108	0.3775	
Paired T/N^b^						
	Paired normal	68	82	6-2344		
	Paired tumour	68	8	0-1855	0.1672	
Location						
	Left colon	22	624	173-1968		
	Right colon	28	719	44-2000	0.899	
	Transcolon^c^	2	154	N/A	0.958	
	Rectum	22	673	175-2522	0.907	
Dukes classification						
	A	7	5539	848-24856		
	B	33	780	104-2272	0.043^d^	
	C	32	611	190-1221	0.015^d^	
Tumour stage						
	T1	2	8000	N/A		
	T2	10	1721	789-10369	1	
	T3	40	639	71-1332	0.226	0.0381^d^
	T4	18	592	215-1600	0.186	
Lymph node involvement						
	Node 0^e^	39	872	213-2874		
	Node 1^e^	16	715	67-1428	0.326	
	Node 2^e^	15	609	200-643	0.045^d^	
TNM staging						
	I	9	2081	814-20038		
	II	30	756	122-2473	0.06	
	III&IV	32	611	190-1221	0.009^d^	
Clinical outcome						
	No invasion	50	835	127-2372		
	Invasion	26	579	161-948	0.1907	
	Disease free	35	759	84-2081		
	Incidence	23	635	217-2187	0.8115	
	No recurrence	58	735	89-2373		
	Local recurrence	7	643	269-3718	0.604	
	No metastasis	50	735	84-2522		
	Metastasis	19	634.3	213-1463	0.92	
	Alive	36	661	86-1876		
	Death	22	572	195-1644	0.923	

^a^Tumour (T), Normal (N). ^b^Transverse colon. ^c^ *p* < 0.05. ^d^Node 0 stands for no node involvement; Node 1 stands for 1 to 3 lymph nodes close to the bowel found to contain cancer cells; Node 2 stands for more than 3 lymph nodes found to contain cancer cells and further than 3cm away from the main tumor in the bowel or the presence of cancer cells in lymph nodes connected to the main blood vessels around the bowel.

**Table 3:** **The correlation of mRNA expression of Kiss-1R and clinical parameters**

Category		No.	Median	IQR	***p***
T/N^a^					
	Normal	80	30	1-2406	
	Tumour	94	<0.000001	<0.000001	<0.0001^c^
Paired T/N^a^					
	Paired normal	68	23	0-522	
	Paired tumour	68	<0.000001	0-0.02	<0.0001^c^
Location					
	Left colon	22	<0.000001	<0.000001-0.01	
	Right colon	28	<0.000001	<0.000001-0.009	0.8791
	Transcolon^b^	2	0.00452	N/A	
	Rectum	22	<0.000001	<0.000001-0.13	0.447
Dukes classification					
	A	7	0.00015	<0.000001-0.00103	
	B	33	0.0004	<0.000001-0.0091	0.6616
	C	32	<0.000001	<0.000001-0.09	0.1431
Tumour stage					
	T1	2	1.06E-05	N/A	
	T2	10	0.00005	<0.000001-0.00171	
	T3	40	<0.000001	<0.000001-0.03	
	T4	18	<0.000001	<0.000001-0.02	
Lymph node involvement					
	Node 0^d^	39	0.0003	<0.000001-0.0071	
	Node 1^d^	16	<0.000001	<0.000001-0.02	0.3236
	Node 2^d^	15	0.01	<0.000001-1.76	0.335
TNM staging					
	I	9	0.00003	<0.000001-0.00033	
	II	30	0.0005	<0.000001-0.0105	0.1939
	III&IV	32	<0.000001	<0.000001-0.09	0.051
Clinical outcome					
	No invasion	50	<0.000001	<0.000001-0.008	
	Invasion	26	<0.000001	<0.000001-0.05	0.1907
	Disease free	35	0.00019	<0.000001-0.00523	
	Incidence	23	0.01	<0.000001-0.12	0.0207^c^
	No recurrence	58	<0.000001	<0.000001-0.013	
	Local recurrence	7	0.00653	<0.000001-0.02245	0.6923
	No metastasis	50	<0.000001	<0.000001-0.009	
	Metastasis	19	<0.000001	<0.000001-0.13	0.0765
	Alive	36	0.00028	<0.000001-0.00724	
	Death	22	<0.000001	<0.000001-0.13	0.0158^c^
	Non treat	42	0.0002	<0.000001-0.006	
	Chemradio	5	0.0796	0.012-0.1607	0.0115^c^

^a^Tumour (T), Normal (N). ^b^Transverse colon. ^c^ *p* < 0.05. ^d^Node 0 stands for no node involvement; Node 1 stands for 1 to 3 lymph nodes close to the bowel found to contain cancer cells; Node 2 stands for more than 3 lymph nodes found to contain cancer cells and further than 3cm away from the main tumor in the bowel or the presence of cancer cells in lymph nodes connected to the main blood vessels around the bowel.

Immunochemical staining was carried out on a portion of paired normal and tumour tissues (n = 23 pairs). Intense expression of Kiss-1 and Kiss-1R was primarily observed in the cytoplasm of epithelial cells in adjacent normal tissues compared with cancer cells (*p* value < 0.05), as shown in Figure 2A and B.

### Knockdown of Kiss-1 and Kiss-1R and the consequent effect on functions of colorectal cancer cells

**Figure 3:**
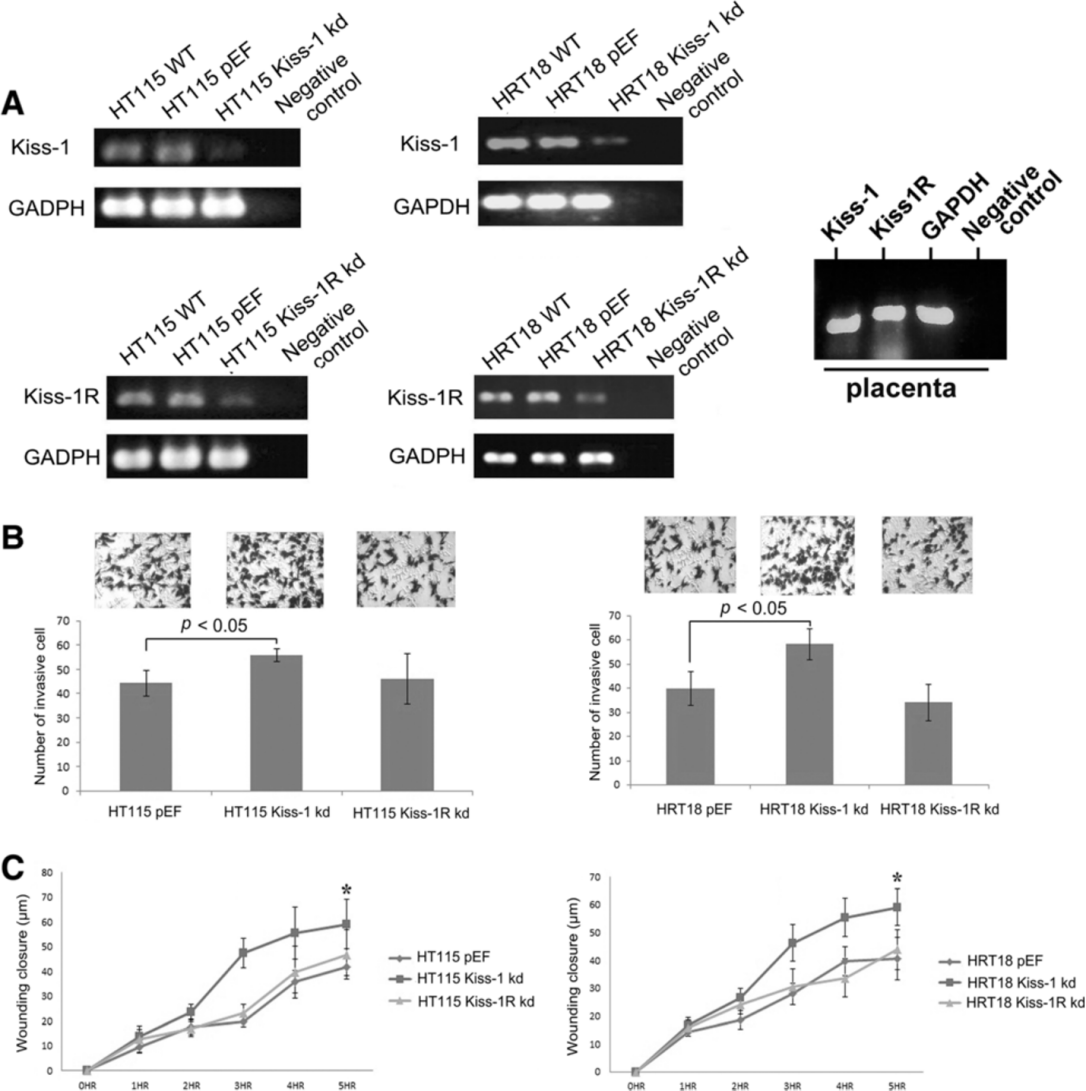
**Knockdown of Kiss-1 altered functions of colorectal cancer cells. A**. RT-PCR displayed a reduced mRNA expression level of Kiss-1 and Kiss-1R in HT115/ HRT18 Kiss-1 knockdown cells and HT115/ HRT18 Kiss-1R knockdown cells compared with control cells (WT and pEF) respectively. Placenta was used as a positive control for both Kiss-1 and Kiss-1R (far right panel). **B**. Left: The influence of Kiss-1 and Kiss-1R knockdown on the invasive capability of HT115 cells (left) and HRT18 cells (right). Three representative images of cells following staining are shown in the top. Data shown is the mean value of 3 repeats of experiment. **C**. The effect of Kiss-1 and Kiss-1R knockdown on the migration ability of HT115 cells (left) and HRT18 cells (right). Data shown is the mean value of 3 repeats of experiment. Error bars represent standard deviation. * stands for *p* < 0.05.

The expression of Kiss-1 and Kiss-1R in both HT115 and HRT18 cells transfected with corresponding ribozyme transgenes was examined using conventional PCR, real time PCR and Western blot (Figures 1 and 3A).

Analysis of the *in vitro* function assays revealed cells with Kiss-1 knockdown had significantly increased invasiveness (*p* = 0.015) and migration (*p* = 0.0094) compared HT115 pEF cells (Figure 3B and C), but knockdown of Kiss-1 and Kiss-1R did not demonstrate a significant change on cell growth and adhesion of HT115 and HRT18 cells (*p* > 0.05).

### Effect of Kiss-1 and Kiss-1R knockdown on the motility of the cancer cells and the involvement of ERK pathway

**Figure 4:**
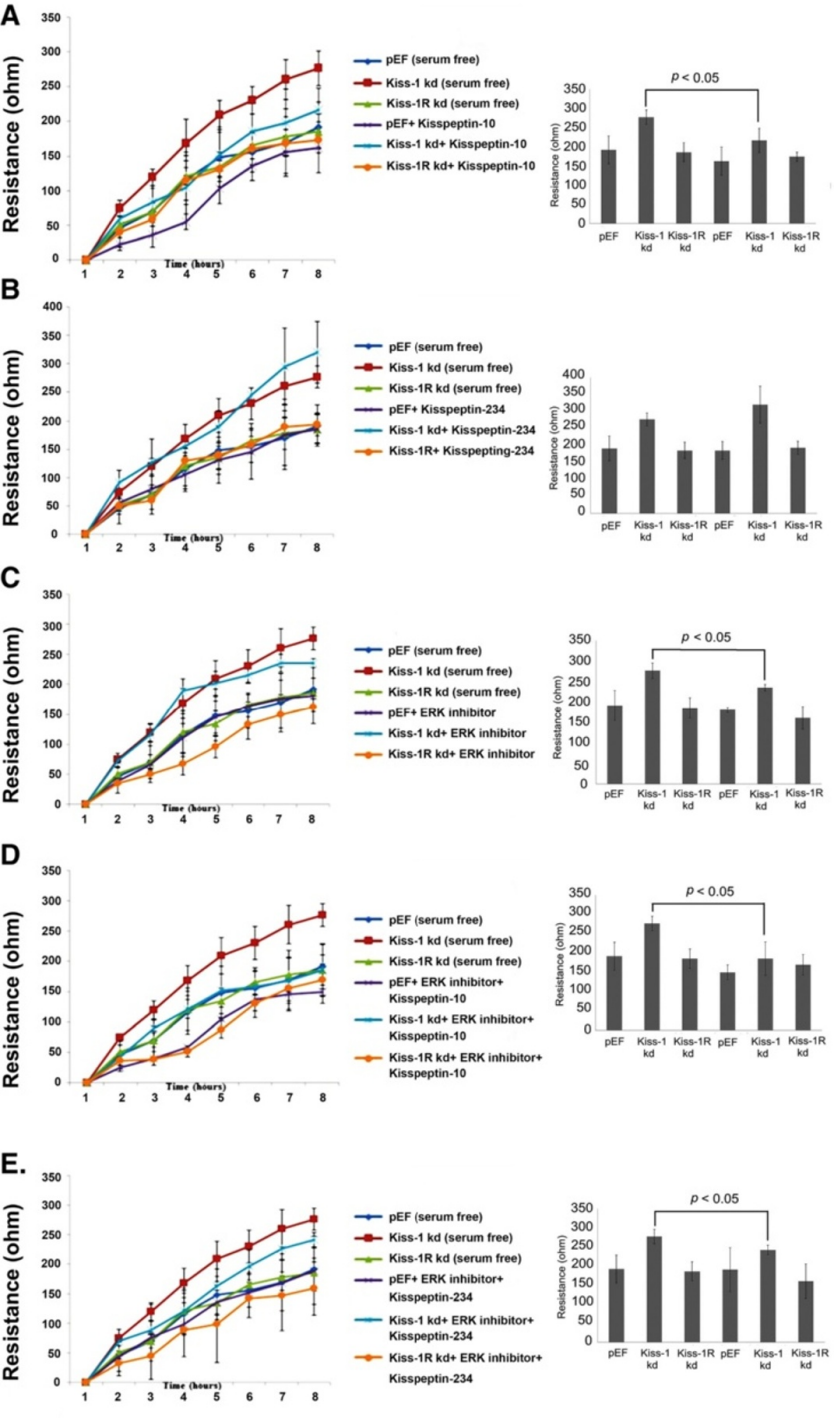
**ERK pathway in the Kiss-1 regulated motility of colorectal cancer cells. A**. Left: The effect of Kiss-1 and Kiss-1R knockdown on the migration ability of HT115 cells treated withKisspeptin-10 compared with control group (no treatment). Right: Overall changes in resistance at the eighth hour * *p* < 0.05 vs the respective control group at the specific time point. **B**. Left: Cells in two different experimental settings (control and Kisspeptin-234) during 8 hours after wounding. Right: Overall changes in resistance at the eighth hour. **C**. Left: The mean trace of the cells in two groups of experiments (control and ERK inhibitor) 8 hours after wounding. Right: Overall changes in resistance at the eighth hour. **D**. Left: Cell responses after wounding (control and ERK inhibitor + Kisspeptin-10) during 8 hours after wounding. Right: Overall changes in resistance at the eighth hour. **E**. Left: Cells responses in different experimental settings, namely control and ERK inhibitor + Kisspeptin-234, over an 8 hours period after wounding. Right: Overall changes in resistance at the eighth hour.

HT115 Kiss-1 knockdown cells treated with Kisspeptin-10 had a significant decrease in motility compared to the control cells (Figure 4A), but no significant difference was observed between control and Kiss-1R knockdown cells. In contrast, neither Kiss-1 knockdown or Kiss-1R knockdown cells treated with Kisspeptin-234 showed a difference in comparison to the control groups (no treatment) (*p* > 0.05) (Figure 4B). The adherence of Kiss-1 knockdown cells treated with ERK inhibitor significantly decreased compared with that of the control group (no treatment) after wounding (*p* = 0.0397) (Figure 4C). A similar effect was also observed in the comparison between the control group (no treatment) and cells treated with ERK inhibitor + Kisspeptin-234 (Figure 4E). In contrast to the groups treated with ERK inhibitor or ERK inhibitor together with Kisspeptin-234, there were more striking links between the Kiss-1 knockdown cells treated with ERK inhibitor + Kisspeptin-10 and its control (no treatment) (*p* = 0.0164) (Figure 4D).

### MMPs are involved in the regulation downstream of Kiss-1/Kiss-1R and ERK pathway

**Figure 5:**
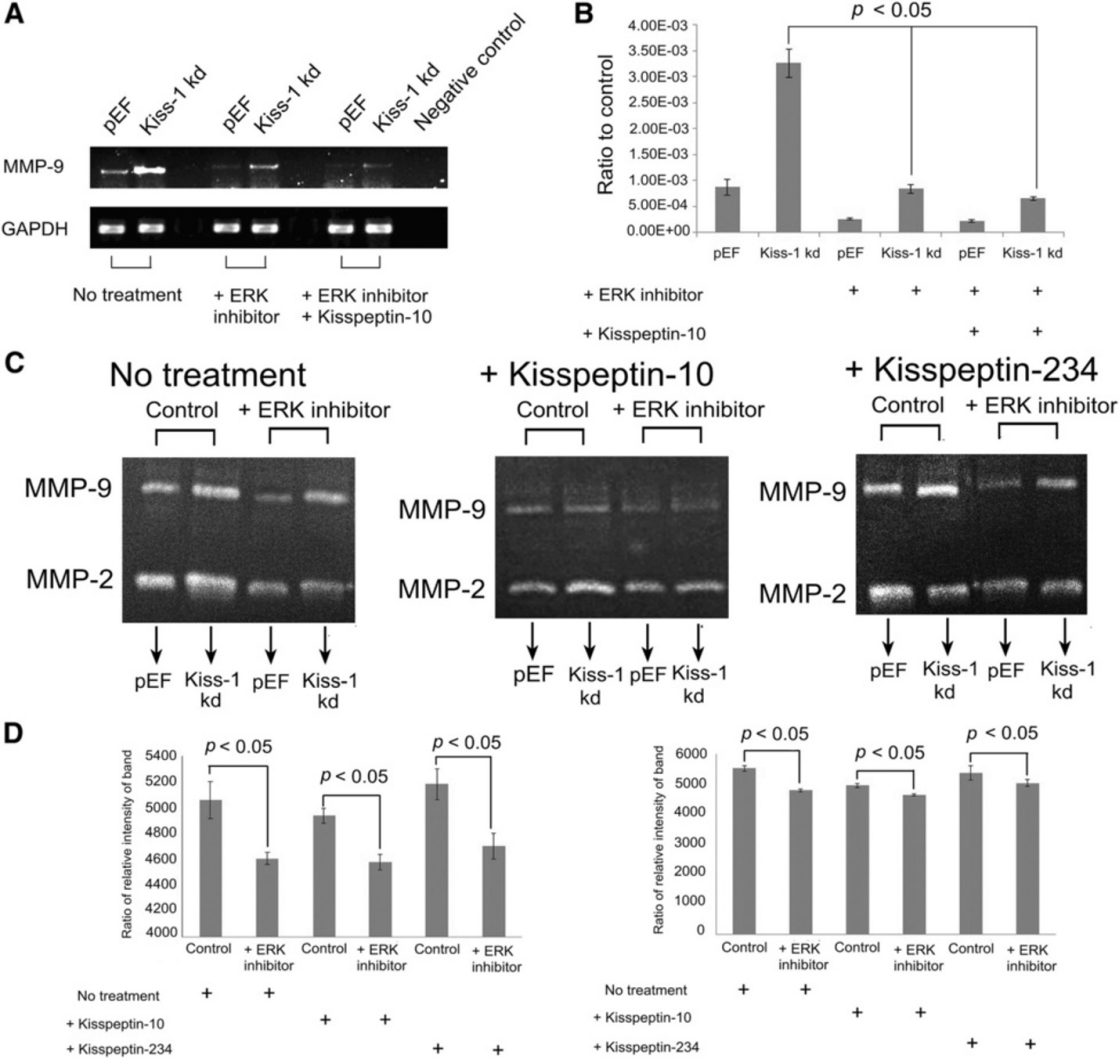
**Kiss-1 expression had an significant impact on MMP-9. A** and **B**. The overall MMP-9 gene expression was increased in HT115 Kiss-1 knockdown cells as shown by RT-PCR **(A)** and by real-time quantitative PCR **(B)**. **C**. The enzyme activity of MMP-9 and MMP-2 in HT115 pEF and HT115 Kiss-1 knockdown cells, following treatment (no treatment, Kisspeptin-10 and Kisspeptin-234). (cells + ERK inhibitor vs control in the group of no treatment: *p* = 0.006577; cells + ERK inhibitor vs control in the group of Kisspeptin-10: *p* = 0.001839; cells + ERK inhibitor vs control in the group of Kisspeptin-234: *p* = 0.005935). **D**. The difference between MMP-9 enzyme activity in three groups of HT115 pEF cells (left) and HT115 Kiss-1 knockdown cells (right). + represents for corresponding treatment. Experiment was repeated three times and mean value taken.

MMP-9 expression levels were evaluated in three pairs of HT115 cells (pEF and Kiss-1 knockdown), which were treated with three different treatments (no treatment, ERK inhibitor, ERK inhibitor and Kisspeptin-10) (Figure 5). The weakest expression of MMP-9 was observed in the cells treated with ERK inhibitor and Kisspeptin-10 (Figure 5A). We further explored the influence of Kiss-1 knockdown together with treatment of Kisspeptin-10 and 234 on MMP-9 and MMP-2 activity, together with the inhibition of the ERK pathway in the HT115 cell line. Cells were divided into three treatment groups, namely no treatment (control group), Kisspeptin-10, and Kisspeptin-234 respectively. For each group, two pairs of HT115 pEF and HT115 Kiss-1 knockdown cells were treated with or without ERK inhibitor, namely + ERK inhibitor and control respectively (Figure 5B). Figure 5C shows expression of Kiss-1 and treatment of the cells with the inhibitors had a greater impact on the enzyme activity of MMP-9 than on that of MMP-2. Hence, the difference in MMP-9 expression in HT115 cells was chosen to analyze further. Figure 5D shows the difference in the enzyme activity of MMP-9 in HT115 pEF (left) and Kiss-1 knockdown (right) cells of all three groups (no treatment, Kisspeptin-10 and Kisspeptin-234). The enzyme activity of MMP-9 in HT115 pEF cells treated with ERK inhibitor was significantly inhibited compared with that of controls in all three groups. Similarly, with HT115 pEF cells, the enzyme activity of MMP-9 in HT115 Kiss-1 knockdown cells treated with ERK inhibitor brought about a strong inhibition in comparison to the controls in the groups of no treatment (*p* = 0.00017) and Kisspetin-10 (0.001544) (Figure 5D). Furthermore, the results show that both HT115 cells treated with Kisspeptin-10 exhibited a decreased expression of MMP-9 in comparison to the cells treated with serum free medium and Kisspeptin-234.

## Conclusions

The expression of Kiss-1 correlated significantly with Dukes classification, TNM staging, tumour stage and lymph node involvement. Interestingly, these stages are classified by the level of tumour invasion and metastasis. Hence, the conclusion from the clinical cohort was consistent with studies in other malignancies. Kiss-1 has a significant, and negative correlation with tumour metastasis and invasion. Loss of Kiss-1R expression was significantly associated with disease free survival, since increased expression of Kiss-1R may have positive correlation with survival. Moreover, high expression of Kiss-1R transcripts was associated with poor prognosis in both overall survival and disease free survival compared with low expression of Kiss-1R. Aberrant Kiss-1 and Kiss-1R expression is definitely a point for consideration. One hypothesis to explain this may be that the inhibitory effect of Kiss-1 on colorectal cancer cells may not solely combine with Kiss-1R, but with other unknown receptors. Navenot *et al.* reported Kiss-1/Kiss-1R also influenced signaling events by interacting with the chemokine receptor (CXCR4) and the gonadotrophin-releasing hormone receptor [17]. Kiss-1R knockdown has little effect on cell migration compared with the control cells which may support this hypothesis. Finally, a recent report has shown that the reduction/loss of Kiss-1 in colorectal cancer may be the result of hypermethylation of Kiss-1, and this provides a plausible explanation for the reduction of Kiss-1 observed in the current study [18]. It will be interesting to explore the mechanisms by which Kiss-1R is reduced/lost in this cancer type in the future.

Data obtained from the present study suggests that Kiss-1 significantly inhibits the motility and invasion of HT115 and HRT18 colorectal cancer cells, but has no significant effect on the proliferation and adhesion of HT115 and HRT18 cells. Interestingly, no increase in cell migration of Kiss-1R knockdown cells compared with the controls was observed. The motility of Kiss-1R knockdown cells would increase the same as the Kiss-1 knockdown cells did, if Kiss-1 indeed had the inhibitory influence on cell migration through Kiss-1R.

Several studies have suggested the mechanism by which Kiss-1 inhibits metastasis may involve extracellular-regulated kinase 1/2 (ERK1/2) phosphorylation and decreased matrix metalloproteinase-2 (MMP-2) expression [11, 19, 20]. In addition, Yan *et al.* reported Kisspeptin-10/Kiss-1R activation can dephosphorylate nuclear factor kappa B (NF-Kappa B), and cause it to dissociate from the MMP-9 promoter. This process ultimately results in a decrease of MMP-9 expression in the placenta [21]. It will indeed be very interesting to examine the degradation of NF-Kappa B in response to Kiss-1 signalling in future studies. As a contribution to this endeavor, we have examined the effects of Kisspeptin-10 and ERK inhibitor on the migration ability of HT115 cells using ECIS assays, and found it to suppress the migration of colorectal cancer cells. Addition of ERK inhibitor can diminish the effect of Kisspeptin-10, suggesting the ERK pathway plays an important role in mediating the signaling of Kiss-1/Kiss-1R.

Kiss-1 significantly inhibits the motility and invasion of HT115 cells. It is clear this action is at least partly mediated by regulating the activity of MMP-9. This study further indicates that the effect on MMP-9 is via the ERK pathway rather than the JNK pathway. In summary, it is suggested Kiss-1 inhibits ERK activation and consequently reduces the enzymatic activity of MMP-9 caused by the degradation of NF-κB, which contributes to the suppression of tumour metastasis.

Perhaps the most intriguing property of Kiss-1 is its potential to be exploited clinically. In the future we are interested in investigating the influence of Kisspeptin-10 on tumour growth and metastasis in an *in vivo* tumour model. Furthermore, Ziegler *et al.* reported that anti-proliferative function of Kisspeptin-10 was regulated by the expression levels of Kiss-1R [22]. There was no effect on proliferation observed in the breast cancer cells expressing Kiss-1R endogenously, while Kisspeptin-10 caused a significant inhibition of proliferation of transfected neuronal cells over-expressing Kiss-1R [22]. Hence, detecting the influence of Kisspeptin-10 on colorectal cancer through changing Kiss-1R’s expression levels will be the focus of follow-up studies.

In conclusion, the present study has presented evidence that Kiss-1 is a putative metastasis suppressor molecule in human colorectal cancer. This is seen by its inhibitory effect on the migration and invasion of colorectal cancer cells, and on actions achieved by down-regulating the activities of MMP9, via an ERK dependent pathway. The preliminary data from studies on a clinical cohort of colorectal cancer patients support this argument.
